# Tartrate-resistant acid phosphatase augments the bone anabolic response to mechanical loading in male mice

**DOI:** 10.1093/jbmrpl/ziaf073

**Published:** 2025-04-23

**Authors:** Bhavik Rathod, Hasmik Jasmine Samvelyan, Suchita Desai, Laura Bock, Nicole Gustafsson, Jianyao Wu, Claes Ohlsson, Per Magnusson, Göran Andersson, Sara H Windahl

**Affiliations:** Division of Pathology, Department of Laboratory Medicine, Karolinska Institutet, 141 52 Huddinge, Sweden; Department of Microbiology, Tumor and Cell Biology, Karolinska Institutet, National Pandemic Center, Stockholm, 17165 Solna, Sweden; School of Medicine, Medical Technology Research Centre, The Faculty of Health, Education, Medicine and Social Care, Anglia Ruskin University, CM1 1SQ Chelmsford, United Kingdom; Division of Pathology, Department of Laboratory Medicine, Karolinska Institutet, 141 52 Huddinge, Sweden; Division of Pathology, Department of Laboratory Medicine, Karolinska Institutet, 141 52 Huddinge, Sweden; Department of Microbiology, Tumor and Cell Biology, Karolinska Institutet, National Pandemic Center, Stockholm, 17165 Solna, Sweden; Department of Internal Medicine and Clinical Nutrition, Institute of Medicine, Sahlgrenska Osteoporosis Centre, Centre for Bone and Arthritis Research, Sahlgrenska Academy at The University of Gothenburg, 413 46 Gothenburg, Sweden; Department of Internal Medicine and Clinical Nutrition, Institute of Medicine, Sahlgrenska Osteoporosis Centre, Centre for Bone and Arthritis Research, Sahlgrenska Academy at The University of Gothenburg, 413 46 Gothenburg, Sweden; Division of Clinical Chemistry, Department of Biomedical and Clinical Sciences, Linköping University, 581 85 Linköping, Sweden; Division of Pathology, Department of Laboratory Medicine, Karolinska Institutet, 141 52 Huddinge, Sweden; Division of Pathology, Department of Laboratory Medicine, Karolinska Institutet, 141 52 Huddinge, Sweden

**Keywords:** ACP5, bone, cartilage, growth plate, growth plate bridges, mechanical loading, TRAP

## Abstract

Tartrate-resistant acid phosphatase (TRAP) is an enzyme predominantly expressed in osteoclasts, where it plays a pivotal role in bone remodeling. Deficiency in TRAP leads to severe skeletal impairments such as osteopetrosis in humans and mice. While mechanical loading is known to promote bone mass and growth, the role of TRAP in this adaptive process remains unclear. Here, we applied axial tibial loading and micro-CT analysis to investigate differences in anabolic response to mechanical loading in 16-wk-old male TRAP KO (TRAP^−/−^) and WT littermate control mice. In WT mice, mechanical loading enhanced the tibial periosteally enclosed area, trabecular bone volume fraction, trabecular thickness, and trabecular number, indicating a robust anabolic response to mechanical strain. In contrast, TRAP^−/−^ mice failed to increase cortical bone and displayed markedly reduced trabecular bone formation under the same loading conditions. Analysis of epiphyseal growth plate bony bridges revealed that the number of bridges in lateral tibiae was reduced in TRAP^−/−^ mice compared to that in WT control mice following mechanical loading, signifying an impaired mechanoadaptive response. Serum alkaline phosphatase concentrations in TRAP−/− mice were similar to those in WT controls, indicating that the inability to respond to mechanical load in TRAP−/− mice is due to TRAP’s specific role in the bone’s adaptive responses to mechanical loading, possibly involving partially impaired osteoblastic bone formation. These findings highlight TRAP as a mediator of bone adaptation to mechanical loading. Understanding the functions of TRAP in mechanoadaptation could direct therapeutic strategies aimed at improving bone strength and treating conditions associated with TRAP dysfunction.

## Graphical Abstract

**Graphical Abstract:**
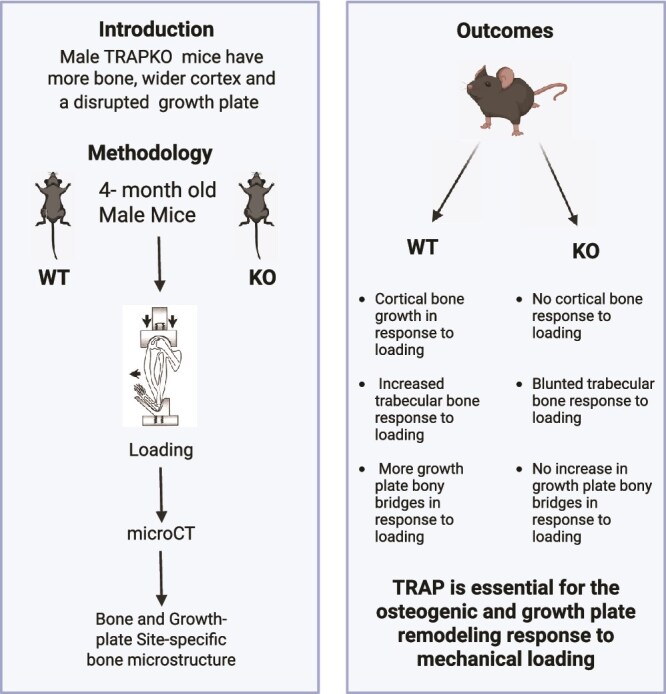

## Introduction

Bone is a highly dynamic tissue serving, both as a structural framework and a reservoir for minerals like calcium and phosphate.^1^^,^^2^ To exert its functions, bone adapts to various stimuli, such as mechanical loading and endocrine hormones, processes that are tightly regulated.^3^^,^^4^ Mechanical loading, for example, through load-bearing physical activity, leads to increased bone mass through a process known as mechanotransduction. In this process, mechanical signals are converted into cellular responses.^4^^,^^5^ These cellular responses trigger adaptive changes where bone is formed or removed where needed to optimize bone architecture, rendering it more efficient in terms of structure and functionality.^4^^,^^6^ In rodents, load-bearing physical exercise can be mimicked by axial mechanical loading of the tibia. In this model, bone formation can be studied independently of bone resorption.^7^ By applying mechanical loading to genetically modified mice or in combination with various drugs, it is possible to investigate the mechanisms whereby mechanical stimuli regulate bone formation in vivo.^4^^,^^8^

One important factor associated with bone is tartrate-resistant acid phosphatase (TRAP), primarily a marker of osteoclasts although it is also present in osteoblasts and osteocytes.^9–11^ It is initially produced as a monomeric polypeptide with low enzymatic activity but can be transformed into a dimeric protein with high enzymatic activity after cleavage by cathepsin K.^12^^,^^13^ TRAP contributes to the balance between resorption and formation, both by playing a direct role in the stimulation of osteoclast migration and indirectly by recruiting osteoblasts for bone formation.^14^ TRAP is also crucial in promoting osteoclast migration during bone resorption by dephosphorylating the bone matrix protein osteopontin and thereby facilitating osteoclast movement. During active bone remodeling, TRAP levels rise in the plasma along with processes such as osteoclast differentiation, activation, and resorption.^15–17^ Furthermore, TRAP plays a crucial role in processes important for skeletal development and remodeling, such as collagen synthesis, dephosphorylation of skeletal phosphoproteins and the release of growth factors that stimulate the proliferation of osteoblasts.^14^ Individuals with inactivation of the TRAP gene due to genetic mutation(s), suffer from spondyloenchondrodysplasia, which is a severe disease with skeletal, immune and neurological manifestations.^18^

Previous studies on TRAP KO mice (TRAP^−/−^) have shown that absence of TRAP protein results in structural changes in bones and growth plates, particularly in male mice.^19^ More specifically, TRAP^−/−^ mice have shorter appendicular bones that are more mineralized than WT mice, and the growth plates appear wider and highly disorganized in comparison to WT control mice.^19^^,^^20^ The growth plates play a critical role in longitudinal bone growth, containing a network of bony bridges spanning across the length of the growth plate that facilitate the transfer of mechanical loads from the knee to the lower limb, which is essential for appropriate skeletal development and stability.^23^^,^^24^ Samvelyan et al. previously reported increased bony bridge formation and anatomical variations in epiphyseal growth plates of mechanically loaded murine models of osteoarthritis.^21^ Interestingly, our recent findings reveal that bony bridge formation is significantly reduced in male TRAP^−/−^ mice, suggesting that TRAP deficiency may affect growth plate dynamics and compromise skeletal stability.^19^ However, the interaction between TRAP and mechanical loading, particularly in relation to bone formation and molecular, cellular processes, and remains unexplored. Further, TRAP overexpressing mice have increased bone remodeling and increased bone mass,^22^^,^^23^ and our previous results revealed increased cortical and trabecular bone mass in male but not female TRAP^−/−^ mice.^19^ Therefore, we here investigated the importance of TRAP for the impact of mechanical loading on bone formation in male mice.

## Materials and methods

### Animals

Sixteen-week-old male WT (*N* = 10) and KO (*N* = 9) mice were obtained from breeding C57BL/6-TRAP heterozygous mice as reported previously.^19^^,^^20^ The mice were housed in groups of up to 5 animals per cage (IVC-500 cages) enriched with paper tunnels, nesting material and softer bedding material (fine chipped saw dust-Tapvei-2HBB175kg) during the loading experiment. The mice were housed in a controlled temperature (22 °C) and photoperiod (12 hr of light, 12 hr of dark), and given water and pellet diet (CRM[P], Special Diet Services, Scanbur) ad libitum*.* All animal experiments were approved by the local Ethical Committees for Animal Research (Ethical number 8387-2018 with amendments) and complied with the ARRIVE guidelines. The animals were euthanized by extraction of blood from the auxiliary blood vessels under ketamine (Abcur) + dexdomitor (Orion Pharma AB) anesthesia followed by cervical dislocation. Genotyping was performed as previously described.^19^ Serum was extracted from the blood using Multivette 600 Serum Gel CAT, 600 μL according to manufacturer protocol (Sarstedt).

### Ex vivo strain measurements

**Figure 1:**
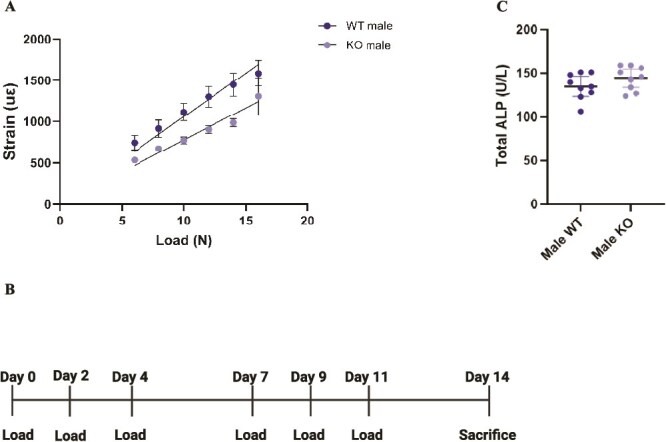
Loading scheme of experiment. (A) Load: strain curve as measured by strain gauge measurements (με) *N* = 5 for WT and TRAP^−/−^. (B) Experimental setup for loading scheme experiment. (C) Total serum ALP levels measured at the end of the study (after loading) were comparable between WT and TRAP^−/−^ male mice. Data displayed as mean ± 95% CI. Unpaired *t*-test with *p*-values indicated in the graph (if significant) *N* = 9 per genotype.

A detailed protocol for ex vivo strain measurements of the mouse tibia used in this study has been reported previously.^24^ In short, the magnitude of axial mechanical strain applied to the tibia during loading was determined ex vivo on euthanized subgroups (*N* = 5 of the experimental groups using a 3100 ElectroForce Test Instrument (TA instruments) to ensure consistent peak strain levels across both groups of mice. Peak compressive loads ranging from 6 N to 18 N were applied to determine corresponding strains (Figure 1B). The same ramping trapezoidal waveform was given to these peak loads, and the identical holding cups and loading instruments were later employed for in vivo loading. A linear regression analysis was performed to analyze the data. To compare the bone loading response between the groups, the mice were subjected to a peak mechanical strain of 1100 με on the medial tibial region corresponding to 10.4 N and 14.2 N for WT and KO mice, respectively.

### Mechanical loading

A detailed protocol for in vivo loading of the mouse tibia used in this study has been reported before.^24^ In brief, mice were positioned so that the knee and ankle joints, held in flexion, were placed into concave cups: the upper cup (containing the knee) was connected to the loading device’s actuator arm, while the lower cup was attached to a dynamic load cell. This setup maintained the tibia under a continuous 0.5 N static preload. Forty cycles of dynamic loading were applied, each followed by a 10-s rest. A peak mechanical strain of 1100 με was used, corresponding to 10.4 N for WT mice and 14.2 N for KO mice. All mice were sacrificed on the third day after the last loading session (Figure 1B).

### Micro-CT

After being removed muscle tissue, tibiae were preserved in 4% formalin for 2 d before being placed in 70% ethanol. Micro-CT analysis was performed on the tibiae using a Skyscan 1275 scanner (Skyscan N.V.) with an X-ray tube voltage of 40 kV and current of 200 μA, together with a 1-mm aluminum filter. The angular increment was 0.4° and the scanning angular rotation was 180°. Every step had an exposure duration of 150 ms. Isomerically, the voxel size was 7 μm. Adaptive local thresholding was used to divide the datasets into binary pictures after they were rebuilt using a modified Feldkamp technique. The proximal tibial trabecular bone was chosen for analysis inside a conforming volume of interest (cortical bone omitted), starting 3% of the bone’s total length distal from the growth plate and continuing further 0.209 μm (30 slices) longitudinally distally from the growth plate. In contrast, the cortical bone analysis was carried out on the entire bone longitudinally using site-specificity analysis. Where sections were analyzed every 1% of the bone length.

### Growth plate bridge analysis

A 3D quantification approach was used to analyze growth plate bridging. To achieve a consistent orientation for growth plate bridging analysis, the scanned and reconstructed images were realigned vertically using DataViewer software (version 1.5.1.2 64-bit, Skyscan, Belgium). The segmentation of micro-CT scans of tibiae was performed using Avizo software (V8.0, VSG). Volume images were manually aligned along the metaphyseal tibial shaft, and the center point of each bony bridge that crossed the growth plate was selected, quantified and projected onto the surface of the tibial joint. The distribution of the areal number density of bridges was superimposed on the tibial joint surface (each bridge has a color that represents the areal number density at the bridge location). From this, the areal number density (*N*, the number of bridges per 256 μm × 256 μm window) was then calculated.

### Measurement of ALP activity

Serum total ALP was measured using a kinetic assay in a 96-well microtiter plate format as reported elsewhere.^25^ In brief, a total volume of 300 μL solution was added per well, containing 1.0 mol/L diethanolamine buffer at pH 9.8, 1.0 mmol/L MgCl_2_, and 10 mmol/L p-nitrophenyl phosphate. The time-dependent increase in absorbance at 405 nm (reflecting p-nitrophenol production) was determined by a Multiscan Spectrum microplate reader (Thermo Electron Corp.).

### Data analysis

SSA was conducted and analyzed as previously described^8^ with statistical analysis performed using linear mixed models in SPSS (IBM, v.22). In short, the bone site was treated as a fixed categorical parameter, while the intervention (loading and genotype) was considered a fixed effect. An interaction between intervention and site was included to account for site-specific responses. Mouse ID was incorporated as a random effect, with repeated measures at different sites for each mouse included in the model. When the overall effect of the intervention was significant, a post hoc Bonferroni correction was applied to pinpoint individual sites where differences were statistically significant. A *p*-value of ≤.05 was considered significant.

The rest of the data are presented as mean ± 95% CI and as individual data points, except when using SSA (site-specificity analysis described above), where comparisons between WT and TRAP^−/−^ mice were made using a repeated-measures ANOVA with a post hoc Bonferroni correction, displayed as mean ± SEM. For other parameters, % changes of loaded vs non-loaded leg were evaluated by paired *t*-tests (WT vs TRAP^−/−^). An effect was considered significant when *p* ≤ .05.

## Results

### The cortical loading response is absent in male TRAP^−/−^ mice

**Figure 2:**
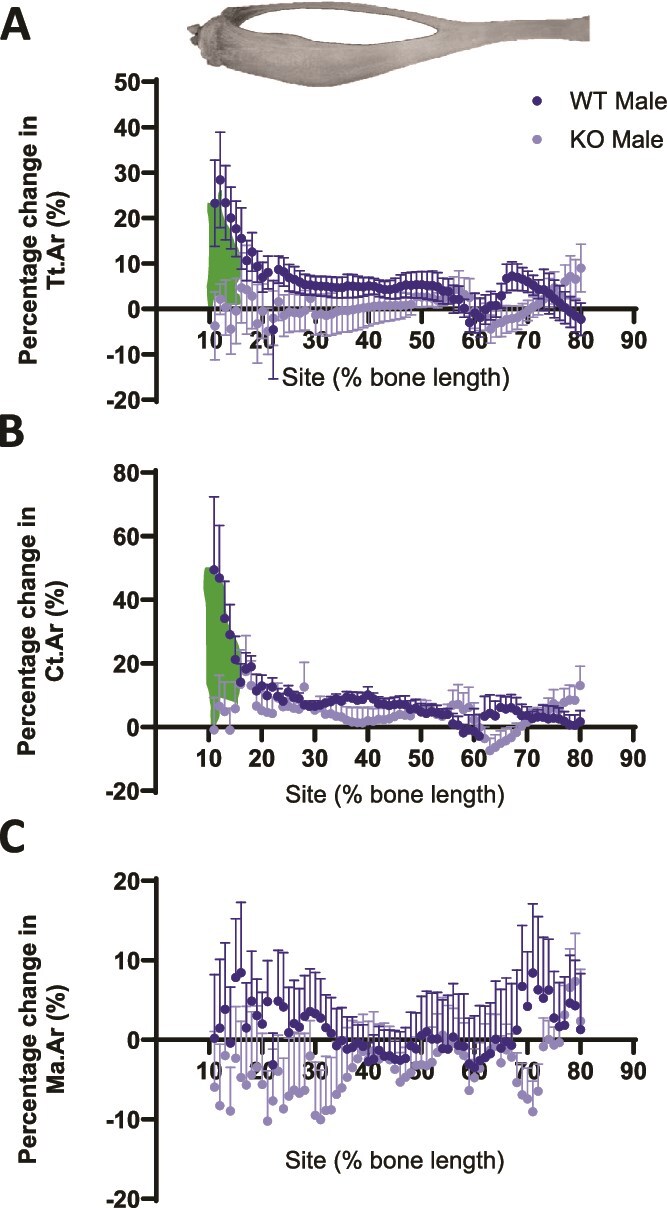
Load-induced cortical bone formation is absent in male TRAP^−/−^ mice. Site-specificity analysis of loading-related percent change in (A) periosteally enclosed area (Tt.Ar), (B) cortical area (Ct.Ar), and (C) marrow area (Ma.Ar). Points represent the mean ± SEM of the percentage change in the loaded vs non-loaded (internal control) tibia of WT and TRAP^−/−^ mice. Shaded region indicates a significant statistical difference of *p* < .05. *N* = 7-10. The representative tibia on top of the graphs schematically indicates the location of the corresponding site on the x-axis.

We performed pre-gauging analysis to investigate the bone strength in WT and TRAP^−/−^ mice prior to loading. Bones from TRAP^−/−^ mice were stiffer (stronger/more load bearing) compared to their WT littermate controls (*p* < .0001, Figure 1A). To investigate the importance of TRAP for the bone-forming effects in cortical bone, we compared the loading response in cortical bone of WT and TRAP^−/−^ male mice using SSA. In WT males, the periosteally enclosed area (Tt.Ar) and the cortical area (Ct.Ar) increased following loading specifically within the first 10%-20% region of the cortical bone (Figure 2A and B). In contrast, in TRAP^−/−^ mice cortical bone area did not increase in response to loading. Loading had no effect on the marrow area (Ma.Ar) in either WT or TRAP^−/−^ mice (Figure 2C).

### The trabecular loading response is abrogated in TRAP^−/−^ mice

**Figure 3:**
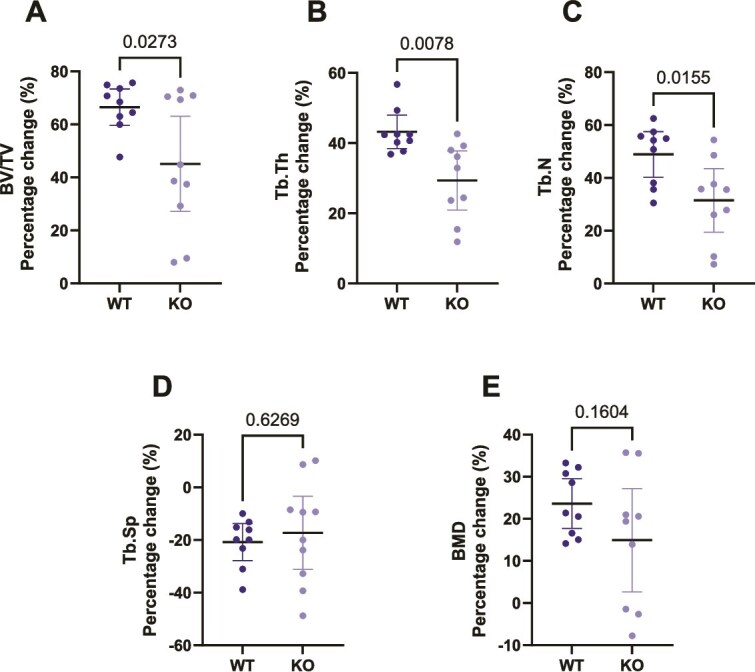
Load-induced trabecular bone formation is reduced in TRAP^−/−^ mice. Micro-CT analysis of trabecular bone. (A) Trabecular volume fraction (BV/TV), (B) trabecular thickness (Tb.Th), (C) trabecular number (Tb.N), (D) trabecular separation (Tb.Sp), and (E) and trabecular BMD. (A-E) Data displayed as mean ± 95% CI. Unpaired *t*-test with *p*-values indicated in the graph *N* = 7-10 genotype. The data is presented as a percentage change in the loaded vs the non-loaded tibia (internal control).

We next investigated the effect of mechanical loading on trabecular bone in both WT and TRAP^−^/^−^ mice (Figure 3). Upon comparing the load-induced percentage changes in BV/TV, Tb.Th, and Tb.N, we observed that WT mice exhibited a significantly greater response to mechanical loading compared to TRAP^−^/^−^ mice, with increases of 67% (*p* = .0273), 43.2% (*p* = .0078), and 48.9% (*p* = .0155) in BV/TV, Tb.Th, and Tb.N, respectively (Figure 3A-C).

In contrast, KO mice showed a diminished anabolic response to mechanical loading, with abrogation in BV/TV, Tb.Th, and Tb.N by −45.1%, −29.3%, and −31.4%, respectively compared to WT mice. Notably, the reduction in trabecular spacing in response to loading was similar in WT and TRAP^−/−^ mice (−21% and −18%, *p* = .6269, respectively, Figure 3D), and BMD remained unaffected by mechanical loading in both genotypes (Figure 3E).

### Load-induced increase in growth plate bridges is reduced laterally in TRAP^−/−^ male mice

**Figure 4:**
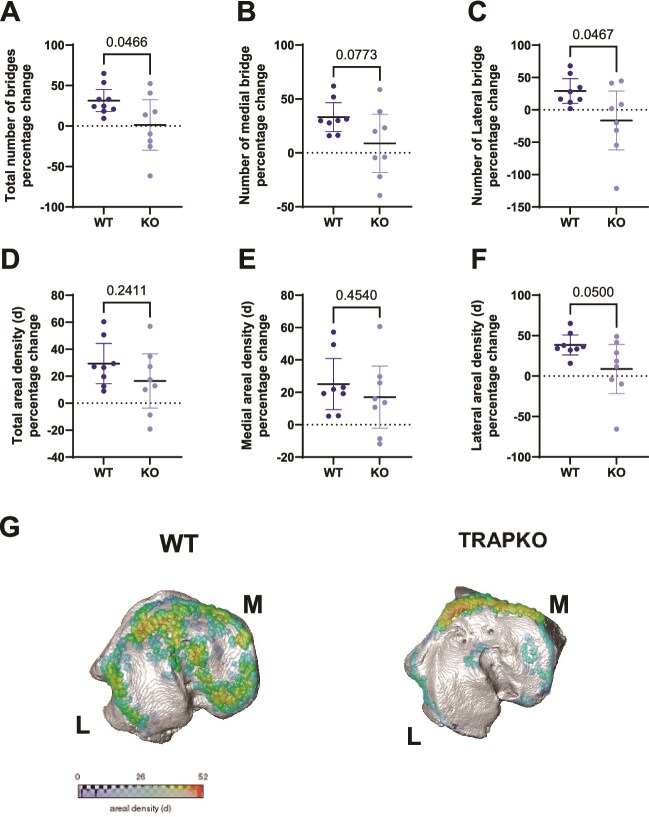
Load-induced increase in growth plate bridges is reduced laterally in TRAP^−/−^ mice. (A) Total number of bridges, (B) number of medial bridges, (C) number of lateral bridges, and (D) total, (E) medial, and (F) lateral areal densities of bridges in tibiae of WT and TRAP^−/−^, male and female mice. (G) Representative images of growth plate bridges of loaded WT and KO mice (unloaded/baseline images are published in Rathod et al.^19^ for comparison). Data displayed as mean ± 95% CI. Unpaired *t*-test with *p*-values indicated in the graph. *N* = 7-10 per genotype. The data is presented as a percentage change of the loaded vs non-loaded tibia (internal control).

We have previously shown that male TRAP^−/−^ mice have a reduced number of growth plate bridges in both medial and lateral sides of the proximal tibia compared to WT mice.^19^ Therefore, we investigated the change in growth plate bony bridges and their areal densities in response to loading. In WT mice, the total number of epiphyseal growth plate bony bridges had a significant increase in response to loading. However, TRAP^−/−^ mice displayed no changes in the total number of bony bridges, largely due to a marked decrease in lateral bridges compared to WT mice (Figure 4A-C). Regarding the areal density of bony bridges, neither genotype showed changes in total areal density in response to loading (Figure 4D and E). However, TRAP^−/−^ mice exhibited a decrease in lateral areal density compared to WT controls (Figure 4F).

### Total serum ALP is normal in TRAP^−/−^ mice

Previously, it was shown that serum P1NP is normal in these TRAP^−/−^ mice. Here, we measured serum alkaline phosphatase (ALP) levels to determine the overall bone formation. No significant differences in serum ALP were observed between the WT and TRAP^−/−^ mice.

## Discussion

Our study highlights the critical role of TRAP in skeletal adaptation to mechanical loading in male mice. TRAP is known to regulate osteoclast function, which plays a central role in bone resorption and homeostasis.^9^^,^^14^^,^^19^^,^^26^ Previous studies have shown that TRAP-deficient male mice exhibit increased bone mass, shorter stature, and a mild form of osteopetrosis, a condition characterized by dense bone mass indicating that TRAP is essential for maintaining normal bone resorption and turnover.^19^^,^^20^ Conversely, TRAP-overexpressing mice demonstrate reduced trabecular bone and a mild form of osteoporosis characterized by microarchitectural deterioration of bone tissue due to increased bone turnover.^22^ Thus, although the importance of TRAP for osteoclast function is well investigated^10^^,^^20^^,^^27^ TRAP involvement in bone formation is yet to be elucidated.^19^^,^^20^^,^^22^

We measured the bone strength of WT and TRAP^−/−^ mice using pre-gauging and found that the tibiae of TRAP^−/−^ mice were less stiff. This is in line with a previous study showing that femur of 4-mo-old TRAP^−/−^ mice are less mineralized than WT mice.^20^ We then used axial tibial loading, a well-validated model for studying bone formation independent of resorption, in young adult male WT and TRAP^−/−^ mice to investigate the role of TRAP in bone mechanoadaptation under controlled mechanical stimuli.^7^^,^^28^ Consistent with earlier reports, WT male mice exhibited an increase in proximal cortical bone size in response to mechanical loading.^8^^,^^29^ In contrast, TRAP^−/−^ male mice failed to show a similar adaptive response, indicating that TRAP is necessary for normal mechanotransduction in bone-forming cells in cortical bone. This impaired response suggests that TRAP may be involved in the regulation of osteoblast differentiation or activation, particularly in response to mechanical strain. This is in line with a previous study showing that TRAP overexpressing mice exhibited an increased bone formation rate.^22^

We also observed significant differences in trabecular bone’s response to loading between WT and TRAP^−/−^ mice. In WT mice, mechanical loading resulted in increased trabecular bone volume fraction, trabecular number, and trabecular thickness, consistent with previous studies indicating that loading enhances trabecular bone parameters.^8^^,^^30^^,^^31^ In contrast, TRAP^−/−^ mice exhibited a markedly blunted trabecular bone-forming response to loading.

In contrast, TRAP^−^/^−^ mice exhibited a markedly blunted trabecular response to mechanical loading. In line with our previous study showing that serum procollagen type I N-terminal propeptide (P1NP) is normal in TRAP^−^/^−^ mice,^19^ serum ALP levels, another well-established marker of osteoblast activity, were normal. Although systemic markers such as P1NP and ALP may not fully capture the site-specific changes induced by mechanical loading, this indicates that the osteoblasts in TRAP^−^/^−^ mice retain the capacity to form bone although these cells could not further enhance bone formation in response to loading. It could be speculated that osteoblasts in TRAP^−^/^−^ mice can form bone, but they have a diminished response to mechanical loading.

Although our study does not include direct cellular analyses in-vitro or by histomorphometery, we would argue that axial mechanical loading was introduced as a model to study bone formation separate from bone resorption and muscle activities, and an increase in bone seen by μCT has been confirmed by dynamic histomorphometery.^28^^,^^32^ Our micro-CT data sufficiently demonstrate that TRAP deficiency compromises bone’s anabolic response to mechanical loading. Anti-resorptive treatment and mechanical loading exert independent beneficial effects on bone,^7^ indicating that osteoblast activity is the main effector underlying the osteogenic effects of mechanical loading. Considering previous work linking TRAP overexpression to increased bone formation,^23^ we speculate that TRAP may modulate osteoblast differentiation or activation in response to strain. Future work is warranted to elucidate the molecular mechanisms underlying TRAP’s involvement in mechanotransduction.

It is important to acknowledge that our use of a global KO model introduces additional complexities. TRAP^−/−^ mice exhibit distinct baseline skeletal properties compared to WT controls, including altered bone geometry and higher bone mass. Although we accounted for these differences by adjusting the actual mechanical loads to generate comparable strain environments, other underlying factors such as altered microarchitecture, systemic signaling changes, or differential strain distribution could not be entirely excluded. Further studies using cell-specific TRAP^−/−^ mice are needed to elucidate the precise contributions of each cell type to definitively establish TRAP’s role in the mechanoregulation of bone.

The process of mechanotransduction in bone cells is primarily facilitated by osteocytes, which serve as mechanosensors by detecting strain and signaling to osteoblasts to promote bone formation.^5^^,^^29^^,^^33^ One may speculate that our findings suggest that the absence of TRAP may disrupt this osteocyte-osteoblast coupling signaling pathway, leading to impaired cortical bone mechanoadaptation to an anabolic stimulus. This is further supported by the fact that TRAP^−/−^ males exhibited wider proximal tibiae, and higher BV/TV, than WT controls, a characteristic typically associated with increased bone formation in response to abnormal strain distribution.^19^ It is possible that the absence of TRAP skews the normal balance between bone formation and resorption, thereby impairing the ability of the skeleton to adapt to mechanical stimuli.

In addition to cortical and trabecular bone responses, our analysis of bony bridges in the growth plate revealed further insights into the role of TRAP in growth plate adaptation to mechanical loading. We observed that WT mice exhibited an increased number of bony bridges in lateral condyle of tibiae following mechanical loading. Interestingly, the load-induced increase in epiphyseal bony bridge formation was absent in TRAP^−/−^ mice. This suggests that TRAP may be required for the proper formation and maintenance of bony bridges under mechanical strain. The lack of an adaptive response in bony bridge number in TRAP^−/−^ mice is particularly striking, as these structures are thought to dissipate mechanical loads and distribute stress across the growth plate.^21^ Thus, the absence of an increase in bony bridges may, at least in part, explain the absence of a loading response in cortical bone, and reduced loading response in trabecular bone seen in TRAP^−/−^ mice. One may speculate that the absence of an increase in bony bridges in response to load could imply that TRAP may be important for adequate mechanotransduction in the growth plate. Further studies are needed to define the precise molecular pathways through which TRAP might regulate the growth plate in response to mechanical strain.

The findings in this study raise several important questions regarding the role of TRAP in bone mechanobiology. While our data suggest that TRAP is important for bone formation in response to mechanical loading, the precise molecular pathways through which TRAP influences mechanotransduction remain unclear. It has been suggested that TRAP may interact with other key signaling molecules involved in bone mechanoregulation, such as the Wnt/β-catenin pathway, which is known to play a critical role in osteoblast activation and bone formation.^33^^,^^34^ The Wnt/β-catenin signaling pathway is a major mediator of osteoblast differentiation and mechanosensation, and disruptions in this pathway have been implicated in skeletal diseases characterized by impaired bone mechanoadaptation, such as osteoporosis. Further studies should investigate whether TRAP deficiency impacts the activity of Wnt/β-catenin signaling or other mechanosensitive pathways in osteoblasts and osteocytes, and whether these effects differ between cortical and trabecular bone.

We acknowledge analyzing key mechanosensitive molecules, such as sclerostin and periostin, would have provided more direct evidence of osteocyte-osteoblast coupling under loading. Sclerostin typically decreases, and periostin increases, in response to mechanical stimuli^34^^,^^35^. Future experiments measuring these markers (eg, via immunohistochemistry or gene expression) could clarify whether TRAP deficiency disrupts these pathways, further elucidating how TRAP contributes to periosteal adaptation and osteocyte mechanosensing. We agree that including both formation and resorption markers would offer a more comprehensive view of bone turnover.

In conclusion, our findings suggest that TRAP plays an important role in bone mechanoadaptation, supporting adequate bone formation under mechanical loading in male mice. The diminished response of male TRAP^−/−^ mice to mechanical stimuli underscores the involvement of TRAP in the mechanotransduction pathway, likely through its impact on osteocyte and osteoblast functions. These findings lay the foundation for further investigations into the molecular mechanisms by which TRAP influences bone mechanobiology.

## Data Availability

The data underlying this article are available in the article.
